# A new species of *Pterygodermatites* (Spirurida: Rictulariidae) in *Marmosa constantiae* Thomas, 1904 from an ecotone area of the biomes Cerrado/Amazon in the Mato Grosso State, Brazil

**DOI:** 10.3389/fvets.2022.955453

**Published:** 2022-09-30

**Authors:** Beatriz Elise de Andrade-Silva, Natália Alves Costa, Richard de Campos Pacheco, Rogério Vieira Rossi, Arnaldo Junior Maldonado

**Affiliations:** ^1^Programa de Pós-graduação em Biologia Parasitária, Instituto Oswaldo Cruz, Fundação Oswaldo Cruz, Rio de Janeiro, Brazil; ^2^Laboratório de Biologia e Parasitologia de Mamíferos Silvestres Reservatórios, Instituto Oswaldo Cruz, Fundação Oswaldo Cruz, Rio de Janeiro, Brazil; ^3^Laboratório de Parasitologia Veterinária e Doenças Parasitárias, Faculdade de MedicinaVeterinária, Universidade Federal de Mato Grosso, Cuiabá, Brazil; ^4^Instituto de Biociências, Universidade Federal Do Mato Grosso, Cuiabá, Brazil

**Keywords:** ecological transitions, marsupials, morphology, Nematoda, Mato Grosso

## Abstract

A new species of nematode, *Pterygodermatites* (*Paucipectines*) *sinopiensis* n. sp. is described based on specimens recovered from the intestine of the white-bellied woolly mice opossum, *Marmosa constantiae*, trapped in the municipality of Sinop, Mato Grosso state, Brazil. The genus *Pterygodermatites* has 21 species described in mammals worldwide, and to date, only two species have been described for marsupials in Brazil. The new species is characterized by the presence of 23 small denticles and by the presence of 38–40 and 65 pairs of the cuticular processes in male and female species, respectively. Additionally, male species possess three ventral precloacal fans, and in female species, the cuticular processes are divided into 41 pairs of comb-like and 24 pairs of spine-like processes; the vulva opens approximately in pair 41. This study describes the parasite species fifth of marsupials in the Neotropical region.

## Introduction

The marsupial *Marmosa constantiae* Thomas, 1904 has a geographic distribution restricted to the central region of South America, occurring in the regions of Bolivia, Argentina, Paraguay, and west-central Brazil (1). It has an arboreal habit, often found in the understory of forests and only occasionally on the ground with an insectivorous–omnivorous feeding habit (2–4).

The Cerrado and Amazon biomes exhibit great biodiversity of small wild mammals but are still little studied (5), as well as their endoparasites, mainly helminths.

The transitional phytogeography of the state of Mato Grosso in the north is formed by ecotones from Cerrado/Amazon biomes and is characterized by two ecotones: seasonal semideciduous forest/ombrophilous forest and seasonal deciduous forest/ombrophilous forest (6).

The first helminth in *M. constantiae* was nematodes belonging to the subfamily Subulurinae Travassos, 1914, genus *Subulura eliseae* (7). The morphological characteristics of the genus *Pterygodermatites* Wedl, 1861 are based on the oral opening position and the total number of cuticular projections, the number of prevulvar cuticular projections in female species, the positions of the papillae on the posterior end, and the size of the spicules in male species (8). The subgenus *Paucipectines* occurs mainly in the Americas and parts of Asia and includes 21 described species found in rodents, marsupials, bats, and armadillos (9, 10).

Here, we report a new species of *Pterygodermatites* parasitizing *M. constantiae*, thus contributing to the inventory of the helminth biodiversity of this host.

## Materials and methods

Fifty-three white-bellied woolly mice were captured in an ecological transition landscape of the Cerrado/Amazon biome from November-December 2016 to June 2017 for eight consecutive nights. Sixteen trapping sessions were carried out with each trapping station consisting of 30 Tomahawk^®^ (16.5 × 16.5 × 35 cm) and 30 Sherman^®^ (9.5 × 8 × 25 cm) traps arranged alternated on soil and the understory (at least 1.5 m high). The traps were baited with a mixture of peanut butter, cornmeal, sardines, bananas, and vanilla flavoring. The collected specimens were anesthetized, sexed, and weighed, and the body size measured. Then, euthanasia was carried out following the procedures established in Resolution No. 1000 of the Federal Council of Veterinary Medicine and Resolution No. 301 of the Federal Council of Biology.

The stomach, the intestine, the lungs, and the thoracic and abdominal cavities of the hosts were searched for helminths. All helminths found were collected and processed according to Hoffman (11) and stored in 70% ethanol. For morphological characterization, specimens were clarified in 50% glycerol, and drawings were produced with the aid of a camera lucida attached to a Nikon Eclipse E200MVR light microscope (Nikon Corporation, Tokyo, Japan). The structures observed using a compound microscope (Zeiss Standard 20) were measured from digital images captured by TCapture Imaging Application software Version 5.1.1.0 (N). All measurements are given in micrometers, with mean ± *SD*, and followed by parentheses for the range (minimum and maximum). The prevalence, abundance, and mean intensity of parasitism of the helminth species found were calculated according to Bush et al. (12).

For scanning electron microscopy (SEM), four specimens (two male and two female species) were processed according to a protocol modified by Souza et al. (13). Then, the samples were critical point dried in CO_2_, mounted on metal stubs, and coated with gold (20 nm). Specimens were examined using a JEOL JSM-6390 LV microscope (JEOL, Tokyo, Japan) at the Rudolf Barth Electron Microscopy Platform Oswaldo Cruz Institute, Fiocruz PDTIS/FIOCRUZ.

## Results

A total of 93 specimens (four male and 89 female species, sex ratios 1/22) of helminths of the genus *Pterygodermatities* were obtained in the small intestine from 16 host specimens among the 53 marsupials examined.

Rictulariidae Railliet, 1916.

**Figure 1:**
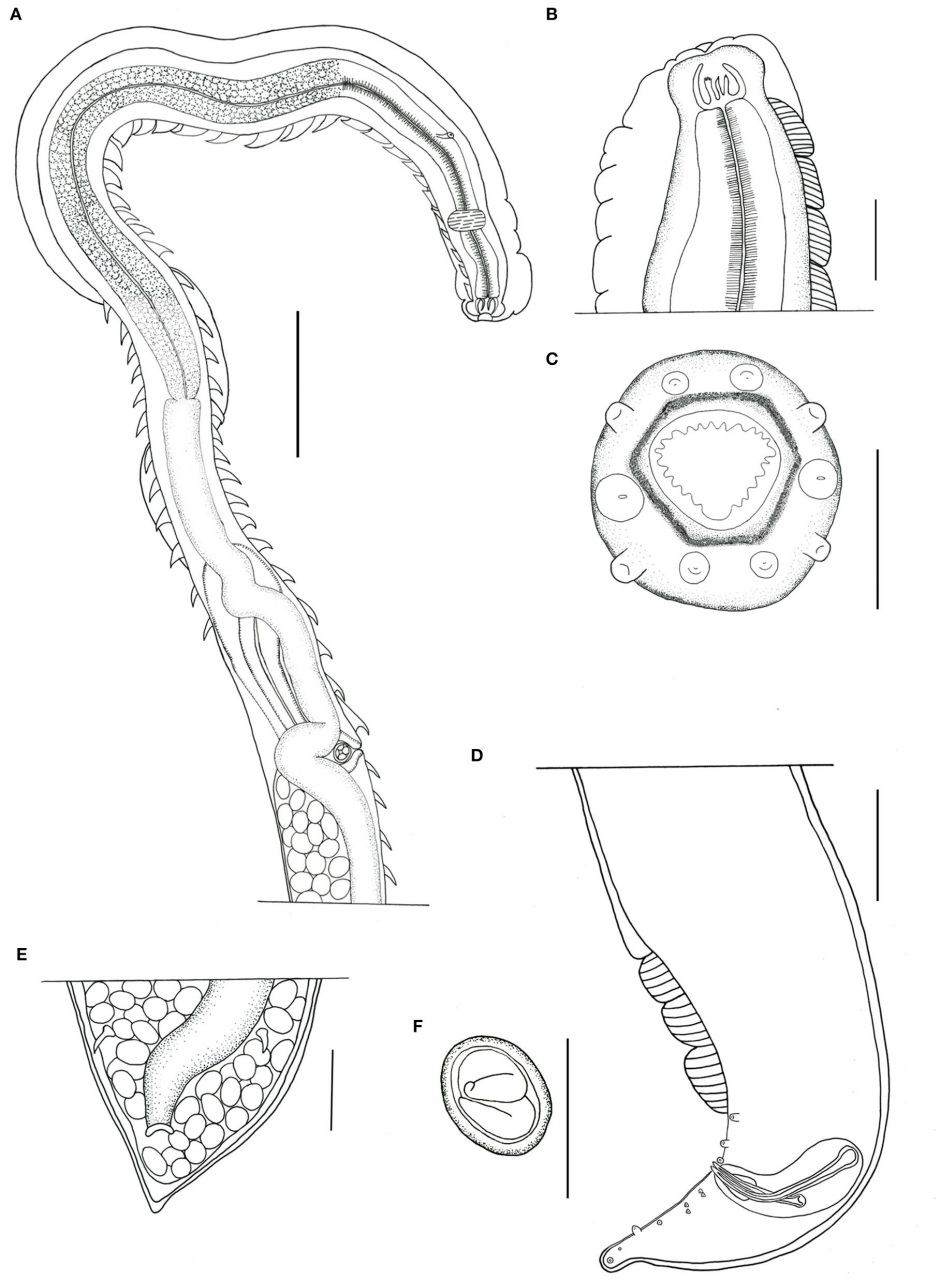
*Pterygodermatites (Paucipectines) sinopiensis* n. sp. **(A)** Anterior extremity, lateral view, female. **(B)** Buccal capsule and esophageal teeth. **(C)** Anterior end, apical view, female. **(D)** Posterior end, left lateral view, male. **(E)** Anus, ventral view, female. **(F)** Egg. Scale = 50 μm **(A,B,D,E)**; 100 μm **(C,F)**.

**Figure 2:**
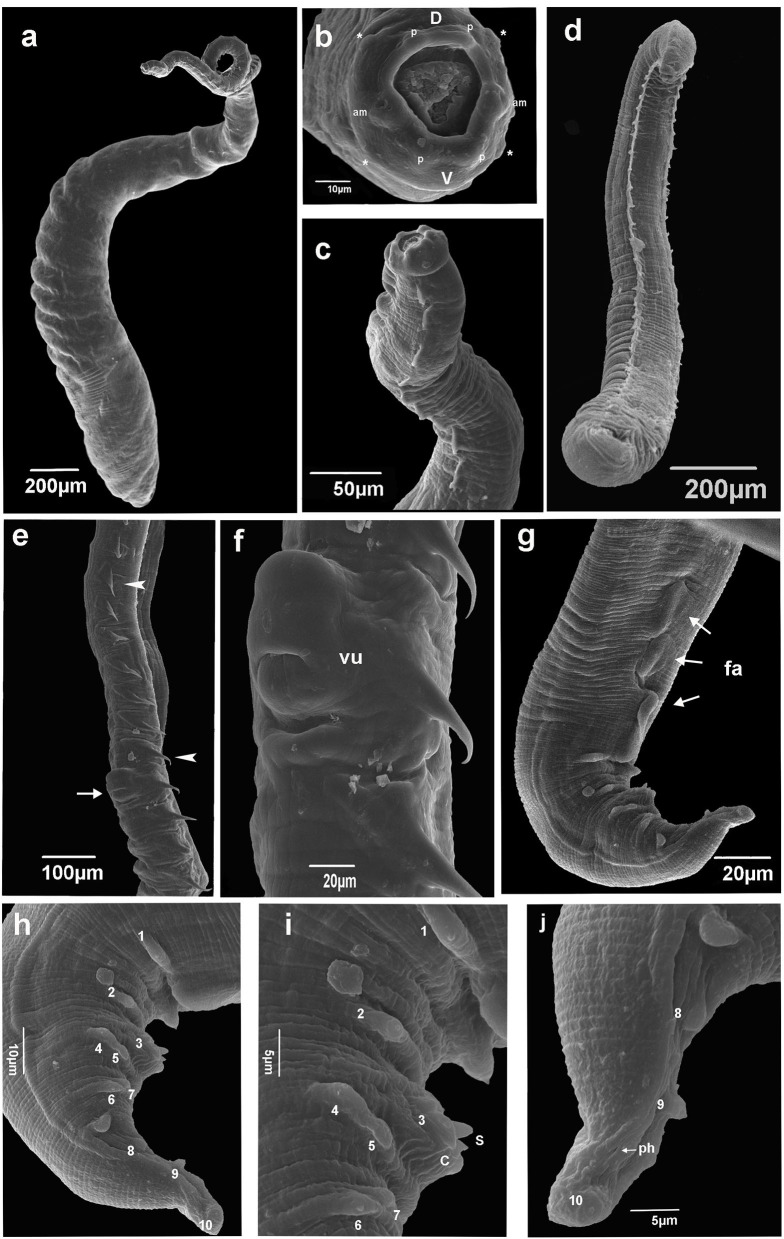
Scanning electron microscopy of *Pterygodermatites (Paucipectines) sinopiensis* n. sp. **(a)** Whole body, female. **(b)** Anterior end is showing oral opening surrounded by small teeth, labial papillae (p), cephalic papillae (asterisk), amphids (am), labial dorsal **(d)**, labials ventrals (V). **(c)** Anterior end, combs lateral view, female. **(d)** Whole body, male. **(e)** Vulva (arrow), spine-like cuticular projections along the female body (arrow head). **(f)** Vulva, ventral view. **(g)** Posterior end, three cuticular fans (arrows, fa) ventral view, male. **(h)** Posterior end, papillae numbered, lateral view, male **(i)** Detail posterior end, tip spicule (S), cloacal open (C), papillae number 1 and 2 precloacal, 3 adcloacal, 4–5 and 6–7 double pair papillae postcloacal male. **(j)** Tail tip, phasmid (arrow, ph), papillae number 8, 9, and 10 postcloacal, male.

*Pterygodermatites* (*Paucipectines*) *sinopiensis* n. sp. (Figures 1, 2).

**Type host:** *Marmosa constantiae* Thomas, 1904; voucher number: UFMT 4,302.

**Site of infection:** Small intestine.

**Type locality:** Sinop (11°49′1.71^′′^S, 55°24′39.05^′′^W), Mato Grosso, Brazil.

**Type material:** Holotype CHIOC: 39370-a; Allotype CHIOC: 39370-b; Paratypes, CHIOC: 39370-c

**Prevalence, mean intensity, and mean abundance:** 30.19%, 5.81, 1.75.

**Etymology:** This species was named according to the locality where the specimens were collected.

### Description

#### General morphology

Small-sized nematodes, with the body slightly widened at the posterior end, with thick cuticles are present. The oral opening is apical asymmetric, with somewhat hexagonal, thick margins, and is surrounded by four pairs of papillae, two cephalic pairs, and two internal labial pairs. Amphids are laterals. Six denticles on each lateroventral margin and 11 denticles on the dorsal margin are present in both sexes. Buccal capsule with thick walls and strongly sclerotized, with three esophageal teeth, the dorsal with irregular edges, and the lateroventral teeth sharp (Figures 1A–C). Two subventral rows of cuticular projections are organized in pairs in both sexes, appearing spine-like along the body, with variable shape, size, and distance from each other, according to their location, starting at end of the buccal capsule and ending at the anus in females and in males anteriorly of ventral fans (Figures 1A, 2a,d). A single type of cuticular projection was present in males (combs), and two different types were present in females: simple spines and combs.

#### Male (holotype and one paratype, two specimens studied by SEM)

The dimension of the body was 2.154 ± 0.53 (1.783–2.526) mm long and 175.5 ± 14.85 (161–182) wide at the esophageal–intestinal junction. Two rows formed 38–40 pairs of subventral cuticular spines distributed parallel along the body length (Figure 2d). Subventral cuticular spines had a width of 40 mm at the esophageal–intestinal junction. The buccal capsule had the following dimensions: 24 ± 2.52 (22–27) long and 20 ± 3.79 (18–25) wide (Figure 1B). The dimension of the total esophagus was 600 ± 106.78 (534–760) length, the muscular portion was 240 ± 93.5 (143–368), and the glandular portion was 359 ± 157.5 (313–551). The nerve ring was located at 141 ± 11.6 (125–148) from the anterior end and the excretory pore, and deirids were not observed, curved ventrally at the posterior end. Ten pairs of basal-like type papillae, arranged as two pairs of precloacal papillae, 1 pair ad-cloacal, and seven pairs post cloacal, the last organized into four pairs of sessile papillae close to the cloacal opening, arranged in two pairs on each side, followed by more three pairs arranged at the same distance to each other, up to the tip of the tail (Figures 2h–j). Phasmids were between the ninth and the tenth pair of papillae (Figure 2j). Three, semicircular cuticular fans were located on the ventral surface of the body, anterior to the first pair of precloacal papillae (Figures 1D, 2g). Spicules were unequal in size, with the left being 108 ± 25.6 (81–111) long and the right being 74 ± 21.1 (53–95) long. Gubernaculum was small, cone-shaped, and poorly sclerotized, with a dimension of 18 ± 1.5 (17–20) long (Figure 1D).

#### Female (allotype and nine paratypes, one specimen studied by SEM)

The dimension of the body was 5.222 ± 0.22 (4.905–5.426) mm in length and 264 ± 38.9 (210–307) in width at the esophageal–intestinal level (Figure 2a). Two rows were formed by 65 pairs of cuticular projections, with 41 being prevulvar and 24 being postvulvar. Subventral cuticular spines of the esophageal–intestinal junction were 56 ± 9.7 (43–73) in width. The first pairs of cuticular spines, anterior to the vulvar opening, were 85 ± 8.6 (71–96) in width (Figure 2a). The buccal capsule was 36 ± 3.9 (30–40) long and 30 ± 4.9 (21–30) wide. The esophagus length was 1.345 ± 95.5 (1.206–1.514) mm, the muscular portion was 279 ± 36.9 (225–309), and the glandular portion was 1.066 ± 81 (910–1.192) mm long. The nerve ring was located 149.5 (*n* = 1) from the anterior end, the excretory pore was 303.6 ± 23.5 (270–333), and deirids were not observed. The vulvar opening was posterior to the esophageal–intestinal junction (Figures 1A, 2e,f). Proeminent vulva elliptic opening was located 2.092 ± 0.10 (1.957–2.273) mm from the anterior end and 601 ± 63.9 (534–707) from the esophageal–intestinal junction, at the level of the 41st cuticular projection. The last spine was located 299.5 ± 35 (235–357) from the posterior end. Opisthodelphic, didelphic, uteri full of embryonated eggs were 31 ± 1.5 (30–34) long and 22 ± 2.4 (22–27) wide (Figure 1F). Conical tail with anus was 174 ± 6.9 (170–190) mm from the tail tip (Figure 1E).

### Remarks

According to Quentin (8), the morphology of the buccal capsule of the specimen described, especially the apical position of the oral opening, enables us to include in the subgenus *Pterygodermatites* (*Paucipectines*). The morphology of the oral opening of the new species is hexagonal, similar to *P. (P.) baiomydis* Lynggaard et al., 2014. The only species with denticles in the range equivalent to a new species is *P. (P.) sibiricensis* Morozov, 1959 (23 vs. 19–24), but the oral opening is rectangular, thus diverging from *P. (P.) sinopiensis* n. sp. Likewise, *P*. (*P*.) *hymanae* Jiménez and Patterson, 2012, *P*. (*P*.) *jagerskioldi* Lent and Freitas, 1935, *P*. (*P*.) *spinicaudatis* Navone and Suriano, 1992, *P*. (*P*.) *peromysci* Lichtenfels, 1970, and *P*. (*P*.) *baiomydis* are distinguished of the new species based on the number of perioral denticles (14, 16, 11–12, 12, and 25, respectively, vs. 23) in *P*. (*P*.) *sinopiensis* n. sp. Although specimens such as *P*. (*P*.) *kozeki* (Chabaud and Bain, 1981), *P*. (*P*.) *dipodomis* (Tiner, 1948), *P*. (*P*.) *argentinensis* Ezquiaga et al., 2017, *P*. (*P*.) *jagerskioldi*, and *P*. (*P*.) *baiomydis*, the *P*. (*P*.) *sinopiensis* n. sp. too have more than 39 prevulvar cuticular processes, arranged in two pairs on each side. Additionally, *P. (P.) sinopiensis* n. sp. can be differentiated from *P. (P.) elegans* (Travassos, 1928), *P. (P.) coloradensis* (Hall, 1916), and *P. (P.) parkeri* (Lichtenfels, 1970), because these three species lack fans and have unpaired caudal papillae, whereas the new species has three fans and all papillae are paired. Moreover, *P. (P.) sinopiensis* n. sp. differs from *P. (P.) microti* McPherson and Tiner, 1952*, P. (P.) ondatrae* Chandler, 1941*, P. (P.) andyraicola* Cardia et al., 2015, *P. (P.) argentinensis, and P. (P.) kozeki* because these species present only one fan, and of *P. (P.) chaetophracti* Navone, 1987 by presents one unpaired precloacal papillae. Two other species, *P. (P.) peromysci* and *P. (P.) onychomis* Cucker, 1939, differ from the new species by the position of the vulva, which in those species is anterior to the esophageal–intestinal junction, whereas in *P. (P.) sinopiensis* n. sp., it is situated 601 μm posterior to the esophageal–intestinal junction. The total number of combs found in females of the species *P. (P.) azarai* (Sutton, 1984), *P. (P.) massoiai* (Sutton, 1979)*, P. (P.) zygodontomis* Quentin, 1967, *P. (P.) jagerskioldi, P. (P.) baiomydis, P. (P.) dipodomis, P. (P.) ondatrae*, and *P. (P.) andyraicola* are much higher than those found in *P. (P.) sinopiensis* n. sp. (65 combs).

## Discussion

Quentin (1969) separated the genus *Pterygodermatites* into five subgenera: *P*. (*Paucipectines*), *P*. (*Neopaucipectines*), *P*. (*Pterygodermatites*), *P*. (*Mesopectines*), and *P*. (*Multipectines*), based on the position of the oral opening, the number of cuticular projections in male and female species, the number of prevulvar cuticular projections in female species, and the geographical and host distributions (8). *Pterygodermatites (Paucipectines) sinopiensis* n. sp. by presenting the morphology of the buccal capsule hexagonal, the apical position of the oral opening, enables us to include them in the subgenus *P. (Paucipectines)*. Although the number of prevulvar cuticular processes (41) is higher than that established by Quentin (29–39) for the diagnosis of the subgenus as well as by Ezquiaga et al. (14), we also propose that the range of this character needs to be revised since other species included in this subgenus also have a high number of prevulvar cuticular processes (8, 14).

In this study, we expand the number of described species in Brazil to four: *P. (P.) elegans, P. (P.) zygodontomis, P. (P.) jagerskioldi*, and *P. (P.) sinopiensis* n. sp. Therefore, the new species is the 22nd species of the subgenus described worldwide. At this time, of the 22 species described for this subgenus, we found parasites of rodents (13), armadillos (2), marsupials (5), and bats (2).

Evaluating the group of host species of the family Didelphidae, we considered that the helminth fauna studied so far is incomplete. Analyzing just the genus *Marmosa* found from Brazil, only four species [*M. demerarae* (Thomas, 1905); *M. paraguayana* (Tate, 1931); *M. murina* (Linnaeus, 1758); and *M. constantiae* Thomas, 1904] have been reported as parasites against five other species [*M. rutteri* (Thomas, 1924); *M. waterbousei* (Tomes, 1860); *M. macrotarsus* (Wagner, 1842); *M. lepida* (Thomas, 1888); and *M. budini* (Thomas, 1920)] without any information about their helminth fauna.

Transition areas often do not receive attention for biodiversity conservation strategies, although these areas may generate responses about the host–parasite relationship. The Cerrado-Amazon transition area is situated in a strongly anthropized area and even in these conditions; it is the habitat of four species of *Marmosa* (*M. budini, M. murina, M. demerarae, and M. constatiae*). In this sense, further investigations into the remaining species of these host groups are necessary to understand the associated parasitological fauna, which could result in the description of numerous new species of helminths, such as representatives of *Pterygodermatites*.

## Data availability statement

The datasets presented in this study can be found in online repositories. The names of the repository/repositories and accession number(s) can be found below: http://zoobank.org/, A86526C1-26BF-42F5-B2C5-163914BE3F64.

## Ethics statement

This study was approved by the Animal Use Ethics Committee (CEUA)-UFMT under protocol number 23108.076870/2015 and the Chico Mendes Institute for Biodiversity Conservation (ICMBio) under protocol number 8863-1.

## Author contributions

BA analyzed and measured the helminths, produced the illustrations, and wrote the manuscript. NC analyzed and measured the helminths and revised the manuscript. RR and RP collected host marsupials and parasitic helminths and revised the manuscript. AJ reviewed all the stages of the study. All the authors reviewed and approved the final version of the manuscript.

## Funding

This study was financed in part by funding a scholarship as provided by the Coordenação de Aperfeiçoamento de Pessoal de Nível Superior (CAPES)—Brasil—Finance code 001. Fundação de Amparo à Pesquisa do Estado de Mato Grosso-FAPEMAT (#477017/2011) and Conselho Nacional de Desenvolvimento Científico e Tecnológico—CNPq (#447557/2014-9; #310352/2016).

## Conflict of interest

The authors declare that the research was conducted in the absence of any commercial or financial relationships that could be construed as a potential conflict of interest.

## Publisher's note

All claims expressed in this article are solely those of the authors and do not necessarily represent those of their affiliated organizations, or those of the publisher, the editors and the reviewers. Any product that may be evaluated in this article, or claim that may be made by its manufacturer, is not guaranteed or endorsed by the publisher.
